# Assessment of Relapse Following Intraoral Vertical Ramus Osteotomy Mandibular Setback and Short-term Immobilization

**Published:** 2010-07-19

**Authors:** Koroush Taheri Talesh, Mohammad Hosein Kalantar Motamedi, Mahdi Sazavar, Javad Yazdani

**Affiliations:** ^a^Tabriz Medical Sciences University, Tabriz, Iran; ^b^Trauma Research Center, Baqiyatallah Medical Sciences University, Tehran, Iran

## Abstract

**Aim:** Relapse is an important issue of concern following operations for mandibular setback. Decreasing the immobilization (IMF) period may play a role in this regard. Usual IMF period ranges from 1 to 2 months. We aimed to assess relapse following a 1-week IMF period. **Materials and methods:** This study aimed to assess 40 purely prognathic patients who had undergone Vertical Ramus osteotomy for mandibular setback. After the release of IMF, guiding elastics were used to direct the mandible to maximal intercuspation for 3 weeks. Relapse was measured from cephalometric radiographs preoperatively and 1 year postoperatively. **Results:** The mean skeletal horizontal relapse after 1 year in 40 treated patients was 0.6 mm. **Conclusion:** The mean skeletal horizontal relapse after 1 year was similar to figures reported for this operation with longer fixation.

Surgery of the mandibular ramus for mandibular excess has been performed since early 1900s.^1^ At present, access to the ramus is almost exclusively via a transoral approach.^1^ Following operations for mandibular setback, relapse is of concern. The immobilization (IMF) period may be an influential factor in this regard. The IMF period ranges from 1 to 2 months. Because subcondylar fractures, unlike fractures of other bones, do not require exact anatomic reapproximation, exact reduction of fracture segments may not be absolutely essential.^2^ The period of immobilization may also be decreased. Thus, we sought to assess relapse following Vertical Ramus osteotomy for mandibular setback and 1 week of IMF.

## MATERIALS AND METHODS

**Figure 1:**
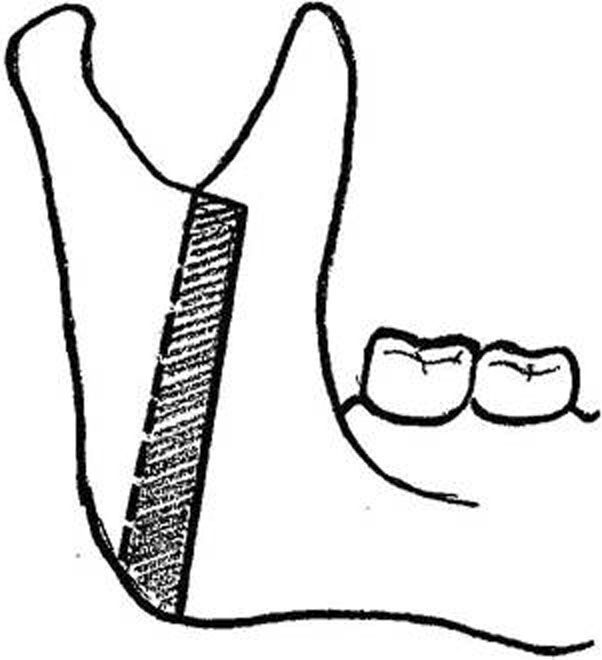
Schematic depiction of the VRO procedure.

This study assessed 40 patients (28 female and 12 male) who had undergone Vertical Ramus osteotomy Fig. 1 and 1 week of IMF for mandibular setback of purely mandibular excess. All had the following criteria:

1. Less than 10 mm jaw relation discrepancy.
2. No open bite.
3. All were adults (end of growth age).
4. Preoperative and postoperative orthodontics.

**Figure 2:**
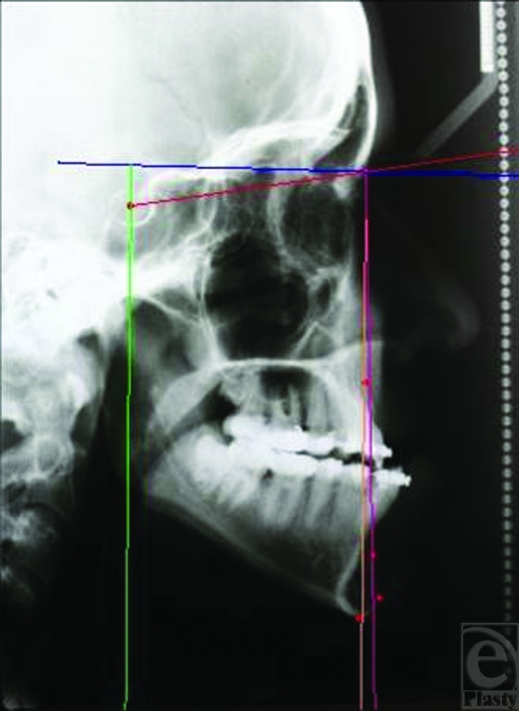
Preoperative cephalogram of a typical prognathic patient.

**Figure 3:**
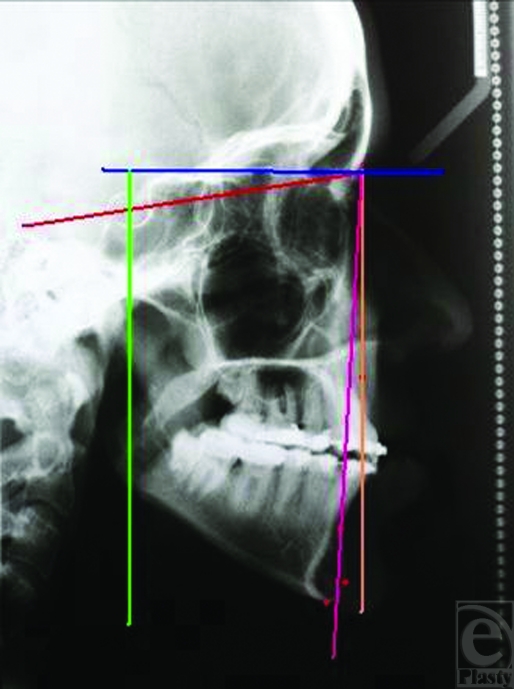
Cephalogram 1-year after surgery.

After the release of IMF, guiding elastics were used for 3 weeks to direct the mandible to maximal intercuspation. Lateral cephalograms were traced preoperatively and 1 year postoperatively. Degree of relapse was measured via sella-nasion-pogonion landmark (Figs. 2 and 3).

## RESULTS

In these 40 patients, the mean setback was 8.9 mm 1 week postoperatively, and after 1 year, it was 8.3 mm, and the mean relapse was 0.6 mm.

## DISCUSSION

The IMF period ranges from 4 to 8 weeks. Following the release of IMF, guiding elastics should be used to direct the mandible to maximal intercuspation for 3 or more weeks.^2^ Relapse is an important issue of concern following orthognathic surgery, especially, operations for mandibular setback.^3^^-^11 In a study by Tornes, there was no significant correlation between postoperative stability and length of osteotomy, and it seems that length of osteotomy is not an important factor in postoperative stability or relapse.^12^,^13^ Mobarak^14^ found that about one-third of the patients, fixed with bicortical screws, showed an anterior postoperative relapse of 2 mm or more. Several authors have described a subcondylar osteotomy, including only the condyle and a small portion of the neck, as a complication of the vertical ramus osteotomy.^3^ The short osteotomy may result in significant rotation and displacement of the proximal segment. This is well-known treatment for TMD.^3^^-^8 Although, various techniques and modifications have been introduced in the treatment of mandibular prognathism, there are still few reports concerning stability, especially using the intraoral vertical ramus osteotomy (IVRO) method. Chen et al^9^ investigated the long-term stability for correction of mandibular prognathism using IVRO. The mean relapse was 1.3 mm (10% of total setback) in a forward direction in his study. The long-term stability of his study suggested that IVRO is useful for correction of mandibular prognathism.^9^ In a study by Hogevold,^4^ 42 patients were operated using the extraoral subcondylar oblique ramus osteotomy and plate fixation; the mean anterior relapse at the 6 months follow-up was 0.5 mm, representing 9% of the surgical setback distance. Yoshioka et al^10^ in 2008 stated that IVRO offers some advantages over sagittal split ramus osteotomy for treatment of the prognathic patient. They concluded that at 1 year after surgery, there was no significant difference between the 2 groups in the stability of the B-point and the pogonion, and that the stability after IVRO is equal to that after sagittal split ramus osteotomy with semirigid internal fixation.^10^ In our study, mean skeletal relapse after 1 year postoperation was 0.6 mm. In other words, 7% of mean total setback. We have yet to see complications such as open bite or malocclusion with this procedure.

## CONCLUSION

The mean skeletal horizontal relapse of intraoral subcondylar osteotomy with short-term immobilization after 1 year was similar to figures reported for this operation with longer fixation.

## Figures and Tables

**Figure 4:**
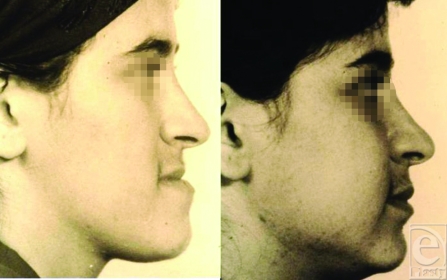
Pre and postoperative photographs.
